# A Roadmap for Building Data Science Capacity for Health Discovery and Innovation in Africa

**DOI:** 10.3389/fpubh.2021.710961

**Published:** 2021-10-11

**Authors:** Joseph Beyene, Solomon W. Harrar, Mekibib Altaye, Tessema Astatkie, Tadesse Awoke, Ziv Shkedy, Tesfaye B. Mersha

**Affiliations:** ^1^Department of Health Research Methods, Evidence, and Impact, McMaster University, Hamilton, ON, Canada; ^2^Dr. Bing Zhang Department of Statistics, University of Kentucky, Lexington, KY, United States; ^3^Department of Pediatrics, Cincinnati Children's Hospital Medical Center, University of Cincinnati College of Medicine, Cincinnati, OH, United States; ^4^Faculty of Agriculture, Dalhousie University, Truro, NS, Canada; ^5^Department of Epidemiology and Biostatistics, University of Gondar, Gondar, Ethiopia; ^6^I-BioStat, Hasselt University, Diepenbeek, Belgium

**Keywords:** big data, health informatics, capacity building, knowledge discovery, data science, Africa, training, stakeholder

## Abstract

Technological advances now make it possible to generate diverse, complex and varying sizes of data in a wide range of applications from business to engineering to medicine. In the health sciences, in particular, data are being produced at an unprecedented rate across the full spectrum of scientific inquiry spanning basic biology, clinical medicine, public health and health care systems. Leveraging these data can accelerate scientific advances, health discovery and innovations. However, data are just the raw material required to generate new knowledge, not knowledge on its own, as a pile of bricks would not be mistaken for a building. In order to solve complex scientific problems, appropriate methods, tools and technologies must be integrated with domain knowledge expertise to generate and analyze big data. This integrated interdisciplinary approach is what has become to be widely known as data science. Although the discipline of data science has been rapidly evolving over the past couple of decades in resource-rich countries, the situation is bleak in resource-limited settings such as most countries in Africa primarily due to lack of well-trained data scientists. In this paper, we highlight a roadmap for building capacity in health data science in Africa to help spur health discovery and innovation, and propose a sustainable potential solution consisting of three key activities: a graduate-level training, faculty development, and stakeholder engagement. We also outline potential challenges and mitigating strategies.

## Introduction

NIH's Strategic Plan for Data Science released in June 2018 (1) defines data science as the interdisciplinary field of inquiry in which quantitative and analytical approaches, processes, and systems are developed and used to extract knowledge and insights from increasingly large and/or complex sets of data (2). The constant evolution of technology in our digital world generated a growing need to discover knowledge and support decision in near real time from large volume of data sets. The versatility, diversity, and connectivity of data capturing devices available today allow data to be generated and stored at increasingly high speed. From national health systems to data collected at rural clinics to the most advanced high-throughput sequencing technologies data are central to our ability to improve health. As data are becoming deeper and richer with new sources of data generated from new technologies and sensors (e.g., social media, geospatial data, mobile phones, wearables, electronic medical records, bioimaging, and genomics), our ability to harness and leverage useful knowledge from these data are critical to accelerate discoveries and innovations that can impact public health (3). Properly harnessed data can provide insights and drive discovery that will accelerate biomedical advances, improve patient outcomes, and reduce costs.

Health Data Science is crucial because traditional study design and analytical approaches are inadequate to tackle challenges posed by the unprecedent volume of large and unstructured datasets. New knowledge generated through the power of data science could enhance precision and patient-focused medicine, cost-effective drug discovery, improvement in patient outcome and delivery of care, as well as support policy makers. Potential applications of data science are increasingly being reported in a wide range of health areas including child health (4), mental health (5), critical care (6), laboratory medicine (7), clinical pharmacology and drug development (8), non-communicable diseases (9–11), physical medicine and rehabilitation (12), and infectious diseases such as COVID-19 pandemic (13).

Some argue that the world's most valuable resource is no longer oil, but data (14). But as oil needs to be refined, data must be properly and optimally analyzed so that it can be transformed into new knowledge. The key question then becomes, how could the availability of data be harnessed so that health innovations and breakthroughs can be achieved and help alleviate sufferings of individuals, communities and society at large, as well as reduce the economic burden on healthcare systems? Addressing this question is urgent in the context of Africa. Like the rest of the world, recent technological advances are enabling African researchers to collect voluminous and complex data at an unprecedented rate on a wide range of health conditions and domains including biomedical, clinical, public health and health systems. However, the ability to harness these data and generate new knowledge is lagging in Africa due to lack of well-trained data scientists.

Although Africa comprises 15% of the world's population, it bears 25% of the global disease burden (15). Africa's population is expected to double by 2050 as the rate of growth is higher than any other continent including Asia and Latin America. The burden of disease both with respect to communicable and non-communicable diseases is striking across the African continent (9–11, 16, 17). The role data science played at a global level in combatting the Covid-19 pandemic–from infectious disease modeling approaches to risk prediction for various subgroups of populations worldwide–cannot be understated. Covid-19 affected almost every nation on earth, but other infectious diseases like malaria, tuberculosis, HIV/AIDS and Ebola continue to be major causes of mortality and suffering in Africa (13).

Billions of dollars have been committed to combat these and many other communicable diseases by various global funding agencies and there is a wealth of data collected over several decades. The whopping increase in population coupled with disproportionate global disease burden requires local talent to investigate context-specific risk factors, discover new knowledge, and produce relevant and timely evidence to impact health practice and policy appropriate to the culture, aspirations and developmental goals of people and governments in the region.

Data science has important implications in achieving the United Nation's Sustainable Development Goals (SDGs) (18). The SDGs highlight that achieving health and well-being for all requires harnessing data and creating new knowledge and innovations in health and other sectors (19). For example, pattern recognition methodologies and tools allow identifying a segment of the population that might be at high risk for developing chronic and non-communicable diseases. Spatial-temporal data science approaches are crucial in detecting “hot spots” and trajectories over time of emerging and re-emerging health problems including communicable diseases across communities and regions. In this paper, we propose a roadmap for developing a strong health data science program in the African context through problem-based graduate-level training activities, faculty development and stakeholder engagement.

## Building Health Data Science Capacity in Africa

Graduate-level degree programs in Health Data Science are gradually emerging especially in European and North American Universities. African Universities, especially those in the East Africa region, lack Health Data Science programs. From a funding point of view, there are encouraging initiatives that are intended to promote the establishment of Health Data Science programs in Africa. One such initiative is the recent announcement by the U.S. National Institutes of Health (NIH) with a significant investment ($58 million) to catalyze data science and health research innovation in Africa (20).

### Training Health Data Scientists

Building modern health data science capacity is feasible in many countries in Africa mainly due to the relatively less expensive infrastructural and training requirements compared to similar activities conducted in laboratory-intensive disciplines, increasing Internet penetration rates, as well as improved access to health-related public databases and open-source software. In recent years, data science programs for public health and biomedical data have started to emerge. Existing Health Data Science programs are mostly at the Masters level with the possibility of pursuing an interdisciplinary PhD degree. However, the standards for the composition of course work and research requirements for a PhD-level rigorous training are still under development in many universities. In launching Health Data Science program, the overarching goals should include: (a) train a cohort of students in Health Data Science that will have the skills to become independent investigators, research leaders, and research collaborators and contribute to Health Data Science research in Africa, (b) faculty development initiatives to strengthen and improve the curricula to match the rapidly changing technological advancement, and (c) participate and create expert hub and networking for groups focusing on data science training, which might cover topics such as core competencies, curriculum sharing, and supporting other similar programs. In addition, the curriculum should reflect the cultural, traditional and language contexts that are relevant to health problems facing Africa.

**Figure 1:**
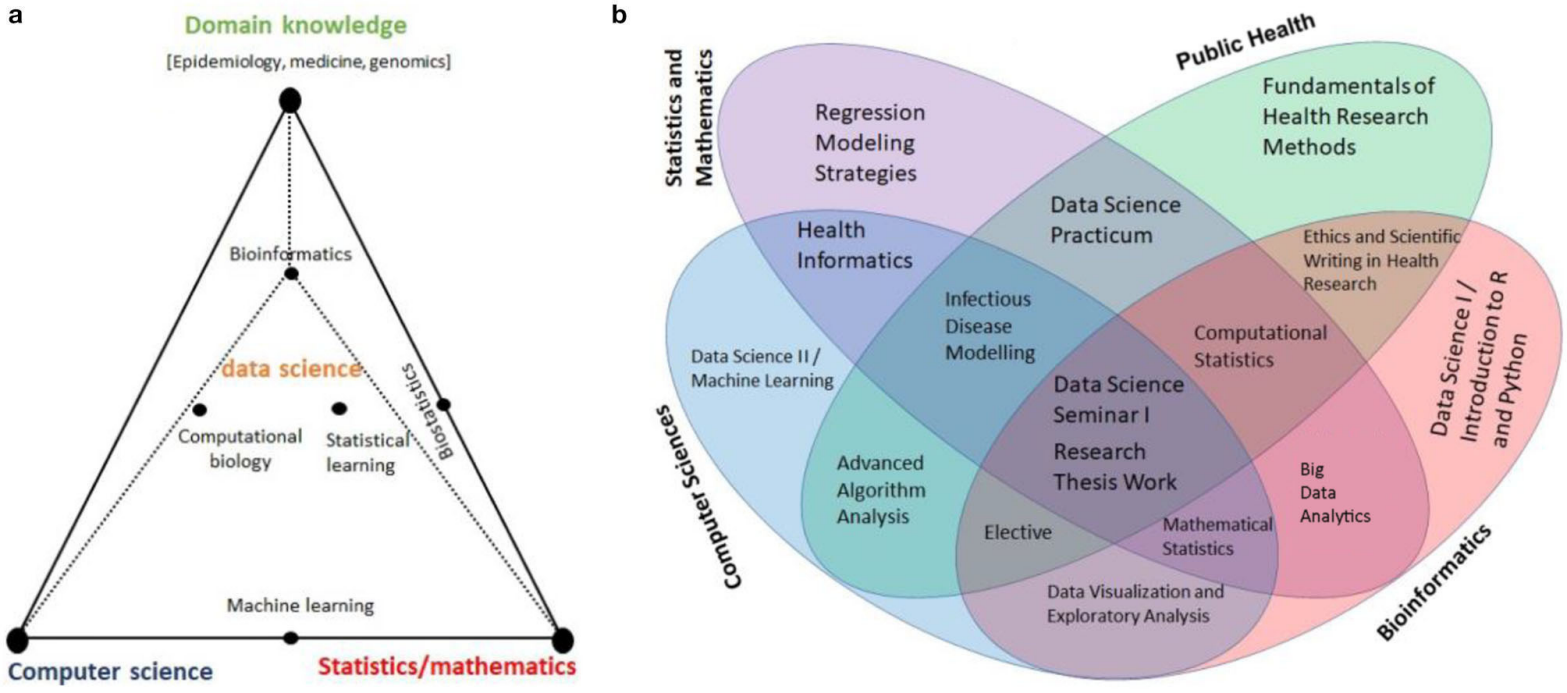
**(a)** Schematic Visualization of the three Pillars: Computer Science/Informatics, Statistics/Mathematics, and domain knowledge expertise [Adapted from (21)], and **(b)** Multidisciplinary Expertise, Skills and Relevant Courses for Health Data Science Program.

The main module of the Health Data Science Training Program should encompass three interdisciplinary areas: (a) Computer Science/Informatics, (b) Statistics/Mathematics, and (c) Domain knowledge experts (21). Figure 1a shows these three pillars along with some examples that combine skills from these focus areas, and Figure 1b displays diverse expertise and skills necessary for a successful Health Data Science training program. The program should include mentors representing varied disciplines with expertise from basic sciences to community-based applied research. There are many African universities that are qualified to host and offer training in Health Data Science. Universities with strong programs in Medical, Biomedical, Public Health, Statistics, Informatics, and Computer Science at MSc, PhD and MD level can serve as the primary hubs to advance training program in Health Data Science in Africa.

The Health Data Science graduate program curricula should prepare prospective students in Africa for careers involving the use of data to inform public-health decision making. The program should involve training in the design, analysis and reporting of health science data, using a blend of traditional and modern analytic and computational techniques. We propose a list of pertinent courses based on which a curricula for graduate programs at various levels can be developed. A set of courses that may be required for acquiring core competency in Health Data Science are listed in Table 1A. These courses spanning health research methodology, statistics and informatics will allow trainees to learn critical skills important in research and application of Health Data Science. In addition to the core courses, a training program should also include a wide range of elective courses (Table 1B) that students can choose from depending on their interest and research focus.

**Table 1:** Proposed Program for Acquiring Competency in Health Data Science (**1A**. Required Courses; **1B**. Elective Courses).

**1A. Required Courses**		
**Statistics/Mathematics**	**Computer Science**	**Public Health**
•Regression Modeling Strategies •Mathematical Statistics •Computational Statistics •Data Visualization and Exploratory Analysis	•Introduction to Computing in Python •Machine Learning •Advanced Algorithm Analysis	•Health Research Methods •Ethics and Scientific Writing in Health Research •Health Informatics •Epidemics Models and Data Science •Infectious Disease Modeling
**1B. Elective Courses**		
•Bayesian Statistics •Causal Inference •Spatial Analysis •Time Series Analysis •Linear Algebra •Numerical Optimization •Theory of Probability •Advanced Calculus	•Natural Language Processing •Big Data Analytics •Artificial Intelligence •Introduction to Cloud Computing	•Fundamentals of Epidemiology •Statistical Genetics •Epidemics Models and Data Science •Computational Biology

### Faculty Development

A diverse and accomplished group of faculty members from various disciplines are crucial for a successful Health Data Science program. The goals of training programs can be achieved and sustained only if institutions invest in the professional development of their faculty members so that they can have successful career in education, research and academic leadership. To address this problem, institutions should create a wide range of faculty development opportunities including customized short-term trainings and workshops for faculty and implement mentorship programs to enhance teaching, research, and service/leadership capacity, and in particular strengthen career development of junior faculty members.

For faculty members to maintain competence and learn about new and developing areas in data science, workshops and short-courses should be designed and offered on a regular basis. Similarly, creating learning opportunities that will enhance scientific writing skills of faculty members, in particular early career researchers, is crucial. The ability to publish research findings in peer-reviewed journals and securing research funding are critical for faculty members to establish and sustain a program of research. Therefore institutions should put a plan in place to help their faculty excel in these important academic activities. Other professional development opportunities may include essentials of supervision, mentoring and leadership foundations.

### Engaging Stakeholders

For a data science training program to be successful, there is a very important and third component–stakeholder engagement. We distinguish two types of stakeholders: (1) domain-knowledge experts who are part and parcel of the data science research team (along with trainees and faculty members), and (2) individuals or organizations who have interest in new knowledge that data science teams generate. To elaborate further, health research will have the greatest impact in every day practice and policy if the model of collaborative research adopts “co-production” of knowledge involving various stakeholders. Similarly, community engagement across scientific disciplines and disseminating findings using various platforms including E-learning is crucial. This process comprises a close collaboration between subject-matter researchers, data science experts, and knowledge users. Health Data Science students should have opportunity to work closely with health researchers who can identify relevant clinical and other scientific problems and have the authority to implement research recommendations. Stakeholders include groups such as clinicians, biomedical researchers, public health experts, health policy makers, community leaders and specific health advocates and support groups. The various groups have unique expertise pertaining to the research topic of interest for their constituencies and knowledge of the context and potential for implementation.

In the collaboration and engagement framework, data science researchers bring methodological and analytical expertise to the collaboration. There are many potential benefits including better science, relevant and actionable research findings, increased use of evidence in policy and/or practice, and mutual learning. Some specific initiatives will include building relationships with knowledge users for implementing data science competence, engage local, national and regional policy makers related to epidemic modeling, as well as link students with people familiar with data hubs so that they can use their data science skills and address important health problems that is relevant to the stakeholders. In addition, the capacity building effort should explore opportunities for engaging data science experts of African origin in the diaspora to harness their teaching and research skills thereby converting brain drain to brain gain.

An E-learning platform could be developed on a minimum budget (22). While the value of courses to in-person attendees is clear and essential, inexpensive access to the internet allows reaching out to a large and geographically dispersed audience, expanding the impact beyond the attendees on campus. The development of an E-Learning platform will help create a collaborative network of users. It should be noted that by E-Learning system we do not mean a “distance learning system,” rather a Web-based platform which offers materials to use in the class for “on campus” courses in Health Data Science. As was proven during the COVID-19 pandemic in many universities across the world, a good and functional E-learning system is essential to ensure the continuation and stability of any educational programs.

## Other Relevant Factors, Challenges and Mitigating Strategies

Here, we briefly highlight additional relevant factors that will be needed to launch and sustain a successful Health Data Science training program. First, trainees should have access to real data sets for hands-on exercises, practicum, and thesis work. Broadly, data could be obtained from two sources: (i) primary data collected by subject-matter experts (biomedical scientists, clinicians, and public health researchers routinely collect primary data to address specific scientific questions), (ii) publicly available data. Supplementary Table 1 (Supplementary Material) lists examples of publicly available datasets. Second, responsible conduct of research must be adhered by all involved in the data science program. Considerable attention should be given to ethics, regulatory issues, scientific rigor, transparency, reproducibility, unbiased and responsible dissemination of research findings, data protection and sharing following well-established guidelines such as the NIH guiding principles of ethical research (23). Third, concrete actions should be taken to promote diversity, equity and inclusivity (DEI) in recruitment, advancement, and retention.

Although Health Data Science promises to advance health discovery, innovations and healthcare delivery in Africa, there are several potential challenges in building the needed capacity. Health Data Science programs require significant investment in infrastructure and human resources. Data collection, storage, management and analysis of big data needs adequate computational facilities including hardware and software, which typically come at a high cost. To mitigate these challenges, public health institutuions, universities, government organizations such as Ministry of Health, and others in the public and private sectors should make efforts to prioritize the budgetary needs of Health Data Science academic programs in their resource allocations. Recruitment and retention of qualified faculty members is another potential challenge in building Health Data Science capacity in resource-limited settings. Brain drain remains to be Africa's largest hurdle for retention of skilled faculty members. The drain is largely driven by lack of incentive mechanisms to recognize the hard work of faculty members and indequate compensations to off-set the consistently rising cost of living. To mitigate this problem, higher education, research and healthcare institutions in Africa should offer competetive salaries and benefits to their employees, including creating opportunities for professional development, promotion, and a conducive environment.

There are many advantages in strengthening capacity locally including the ability to scale up and reduce the possibility of brain drain. It is also vital that trainees be embedded within the environment where they can fully understand and appreciate unique challenges to the local context, culture and other norms. Data science is inherently team-based, so having trainees work closely with people who generate biomedical, clinical, and public health questions will allow them to learn about important problems, gain a broader understanding of the research enterprise, and become all-rounded data scientists. Training locally will also lead to a sustainable solution to the chronic lack of capacity we currently see in Africa. Other important considerations include the advantage trainees will have by not going too far from their community and family, as well as be part of a group that will establish a strong infrastructure and research culture in their home countries.

## Summary and Conclusion

In this paper, we proposed a framework to guide how to build Health Data Science capacity in Africa using three major activities: (1) training health data scientists at a graduate level, (2) faculty development, and (3) stakeholder engagement. These three activities span the entire research process required for addressing health problems in an integrated and collaborative setting. The process encompasses key research components including asking relevant questions, planning appropriate study designs, developing measurement instruments, collecting data, conducting optimal analysis and dissemination of findings. The proposed Health Data Science training program follows a holistic and team-based approach to solving scientific problems related to health: competencies that trainees in the program will acquire, the key role faculty members will play in problem-solving while junior faculty are being empowered to develop their career, and engaging stakeholders to help define important and context-based health problems as well as implement health innovations (24). Solving important problems requires fostering a collaborative environment and involving various team members at different steps of this cycle with a shared vision and dedication to discovering new knowledge and advancing health innovations (Supplementary Figure 1, Supplementary Material).

In conclusion, data science is a rapidly evolving multidisciplinary field which has an important role to play in health discovery and innovations. As technology continues to advance, and big and diverse data become common, the evolving field of data science has the potential to provide the opportunity to create a better future for human health by harnessing these data. Unfortunately, most African nations may not reap the benefits of data science due to lack of well-trained data scientists. This lack of capacity must be addressed urgently for Africa not to continue falling so far behind other parts of the world in this important and promising field of science. Data science requires combining rigorous study design with appropriate statistical inference and computational approaches. Scientific and/or clinical domain knowledge is a key ingredient to harness the potential of data science methods and tools. Finally, perhaps more than other multidisciplinary disciplines, critical skills including effective communication and other team science skills, as well as ethical and responsible conduct of research are necessary in Health Data Science.

## Data Availability Statement

The original contributions presented in the study are included in the article/Supplementary Material, further inquiries can be directed to the corresponding authors.

## Author Contributions

TM, JB, and SH conceptualized the study and drafted the manuscript. All authors critically reviewed the manuscript and approved the final manuscript as submitted.

## Funding

JB acknowledges partial support by the Natural Sciences and Engineering Research Council (NSERC) of Canada, grant RGPIN-2009_293295. JB holds the John D. Cameron Endowed Chair in the Genetic Determinants of Chronic Diseases, Department of Health Research, Methods, Evidence, and Impact, McMaster University. TM acknowledges partial support by the National Heart, Lung, and Blood Institute (NHLBI), grant R01 HL132344.

## Conflict of Interest

The authors declare that the research was conducted in the absence of any commercial or financial relationships that could be construed as a potential conflict of interest.

## Publisher's Note

All claims expressed in this article are solely those of the authors and do not necessarily represent those of their affiliated organizations, or those of the publisher, the editors and the reviewers. Any product that may be evaluated in this article, or claim that may be made by its manufacturer, is not guaranteed or endorsed by the publisher.
