# Supplementary material for: A Roadmap for Building Data Science Capacity for Health Discovery and Innovation in Africa
Source: Front Public Health. 2021 Oct 11;9:710961. doi: 10.3389/fpubh.2021.710961 (PMC8544798; doi:10.3389/fpubh.2021.710961)

Supplementary Material

1 Supplementary Tables and Figures

Supplementary Table S1. Publicly Available Datasets Relevant to Health Data Science Program

Epidemiology and public health related	Genomics, microbiome, multi-omics related
**HCUP:** Healthcare and Cost Utilization project collects an impressive number of data sets deep in quality and depth every year. Here are the most prominent ones.<br>**a.** NIS (National Inpatient Sample). The USA has more than 3000 hospitals. Every hospital collects data on each of its admissions. Information on about 200 variables is collected. HCUP collects a random sample of 20% admissions annually from every hospital and creates a data base, which is named NIS. It has yearly data dating back to 1988. The current year's data is for 2017. One column in the data gives information for what medical condition the patient is admitted into the hospital.<br>**b.** KID (Kid's Inpatient Database). The data refers to pediatric hospitals	**H3Africa**: Genetics data – Human Heredity and Health in Africa. H3Africa is a major program initiated in 2010 by the National Institute of Health (NIH), Wellcome, and African Society of Human Genetics (AfSHG). H3Africa supports population-based studies that use genetic, clinical and epidemiological tools to better understand how the interplay between human genes and the environment in disease susceptibility, pathogenesis and prevention with the goal of improving the health of African populations.
**NHANES** (National Health and Nutritional Examination Survey). The survey examines a nationally representative sample of about 5,000 persons each year. These persons are located in counties across the country, 15 of which are visited each year. The NHANES interview includes demographic, socioeconomic, dietary, and health-related questions. The examination component consists of medical, dental, and physiological measurements, as well as laboratory tests administered by highly trained medical personnel.	**dbGap (Database of Genotypes and Phenotypes).** It is a compilation of several data sets at individual level. Access to any specific dataset is granted upon request with a valid proposal.
**NHIS** (National Health Interview Survey). About 30,000 households are interviewed annually asking questions about health and family dynamics. The survey includes data on about 9000 children.	**African Pathogen Genomics Initiative data**: The Africa CDC Institute for Pathogen Genomics (IPG) is a continent-wide leadership initiative established in 2019 to support public health pathogen genomics and bioinformatics activities in Africa.
**NYTS** (National Youth Tobacco Survey). This is an annual survey of tobacco use among middle and high school students.	**TCGA** (The Cancer Genome Atlas program). The Cancer Genome Atlas (TCGA), a landmark program, molecularly characterized over 20,000 primary cancer and matched normal samples spanning 33 cancer types. This joint effort between the National Cancer Institute and the National Human Genome Research Institute began in 2006, bringing together researchers from diverse disciplines and multiple institutions.
**NPCR** (National Program for Cancer Registries). Every cancer case in the USA is recorded in the registry with detailed information.	**TOPMed**. The Trans-Omics for Precision Medicine (TOPMed) data is a great resource to leverage multi-omics data related to heart, lung, blood, and sleep disorders.
**COVID-19 data** Daily infections by country – Johns Hopkins University COVID-19 Center	**UK biobank.** UK Biobank is among the world's largest repositories for phenotypic and genotypic information in individuals of primarily European ancestry. It is an open-access resource comprised of a population-based prospective cohort of 500,000 men and women aged 40 to 69 years who were registered with the National Health Service (NHS) and resided within the UK between 2007 and 2010.
**CDC-** Data.CDC.gov is a repository of all available data sets with a Socrata Open Data API. Available categories include: Administrative, Biomonitoring, Child Vaccinations, Flu Vaccinations, Health Statistics, Injury & Violence, Motor Vehicle, NCHS, NNDSS, Pregnancy & Vaccination, STDs, Smoking & Tobacco Use, Teen Vaccinations, Traumatic Brain Injury, Vaccinations and Web Metrics	**All of Us**: The All of Us Research Program is initiated to collect and study data from one million or more people living in the United States. The goal of the program is better health for all of us.
**DHS -** Demographic Health System (archive@dhsprogram.com)	

Supplementary Figure S1. An iterative Health Data Science training program involving trainees, faculty, and stakeholders following PPDAC (Problem, Plan, Data, Analysis, Conclusion) approach (adapted from Spieglehalter, 2019).[24]

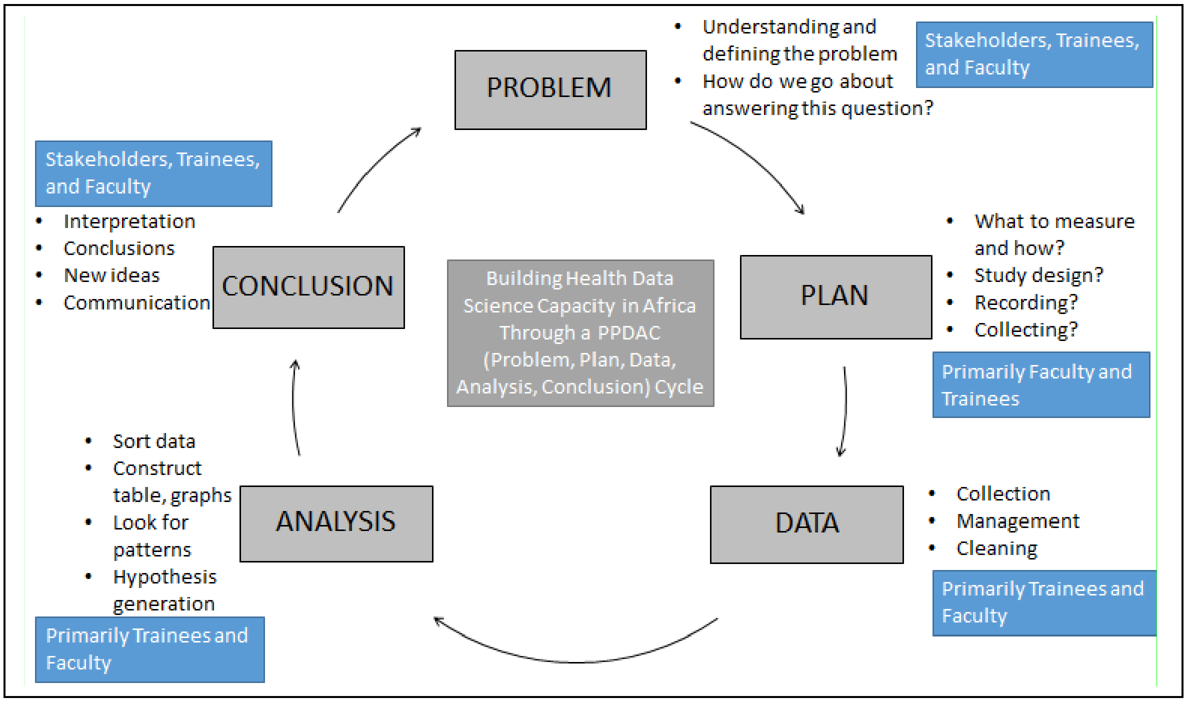

Supplement: Supplementary file 1 [file Data_Sheet_1.pdf]
